# TRANSITIONING TO HOSPICE DURING THE COVID-19 CRISIS: LESSONS FOR CARE COORDINATION AT THE END OF LIFE

**DOI:** 10.1093/geroni/igad104.0625

**Published:** 2023-12-21

**Authors:** Emily Franzosa, Patricia Kim, Laura Moreines, Jonelle Boafo, Daniel David, Abraham Brody, Margaret McDonald, Melissa Aldridge

**Affiliations:** Icahn School of Medicine at Mount Sinai, New York City, New York, United States; Icahn School of Medicine at Mount Sinai, New York City, New York, United States; New York University, New York City, New York, United States; Rory Meyers College of Nursing, New York City, New York, United States; NYU Rory Meyers College of Nursing, New York City, New York, United States; NYU Rory Meyers College of Nursing, New York City, New York, United States; Visiting Nurse Service of New York, New York City, New York, United States; Icahn School of Medicine at Mount Sinai, New York City, New York, United States

## Abstract

The COVID-19 pandemic caused widespread delays and disruption in end-of-life services. We examined the impact of these disruptions among patients in a large, NYC health system who were referred to hospice and died between March 1, 2020 and March 1, 2021. We linked the electronic health records (EHR) of 124 patients cared for by a geriatric outpatient practice to the hospice EHRs to create longitudinal trajectories of patients’ care. We conducted an in-depth qualitative thematic analysis of linked medical and hospice records to examine provider and patient experiences as reflected in the EHR. Our sample (mean age of 91 years) were enrolled in hospice for an average of 173 days, and 81% died at home. We found challenges transitioning from medical to hospice care, including referral and enrollment delays due to infection prevention protocols, communication issues, staffing and equipment shortages (e.g., oxygen, morphine). These disruptions impacted quality of care as patients experienced shortages in personal care services, medical equipment and supplies, and missed home visits due to fear or isolation protocols. Virtual visits helped maintain access to hospice, particularly social work and spiritual care, as did ongoing communication with both the primary care and hospice teams. Our findings demonstrate that while COVID-19 added care transition challenges, new flexibilities and adaptations helped manage disruptions. Retaining these practices, such as reimbursing telehealth social work and spiritual care visits and maintaining ongoing patient contact with trusted primary care providers may help extend emotional support to patients and families during the end of life.

